# Numerical Simulation of Nonlinear Pulsatile Newtonian Blood Flow through a Multiple Stenosed Artery

**DOI:** 10.1155/2015/628605

**Published:** 2015-11-08

**Authors:** Satyasaran Changdar, Soumen De

**Affiliations:** ^1^Institute of Engineering & Management, Saltlake, Kolkata 700101, India; ^2^Department of Applied Mathematics, University of Calcutta, 92 Acharya Prafulla Chandra Road, Kolkata 700009, India

## Abstract

An appropriate nonlinear blood flow model under the influence of periodic body acceleration through a multiple stenosed artery is investigated with the help of finite difference method. The arterial segment is simulated by a cylindrical tube filled with a viscous incompressible Newtonian fluid described by the Navier-Stokes equation. The nonlinear equation is solved numerically with the proper boundary conditions and pressure gradient that arise from the normal functioning of the heart. Results are discussed in comparison with the existing models.

## 1. Introduction

At present the investigation of blood flow analysis in a stenosed artery is very important in the medical domain because of the fact that many of the diseases such as heart attacks and strokes are related to blood flow and the physical characteristic of vessel wall. Nowadays the leading causes of the death in the world are due to heart diseases such as atherosclerosis. Atherosclerosis involves an accumulation of low-density lipoprotein in the wall of large arteries, typically where the wall shear rate is low and oscillatory [1].

Investigation of blood flow modeling through arterial multistenosis is very challenging. Accuracy of the simulation depends mainly on suitable numerical approach, realistic model geometry, and boundary conditions. Many investigators have focused their attention on blood flow through stenosed arteries with single stenosis by Mekheimer [2, 3], Chakravarty and Mandal [4], Lee and Xu [5], who pointed out that the mathematical model becomes more accurate in the presence of an overlapping stenosis instead of a mild one. Ang and Mazumdar [6] studied asymmetric arterial blood flow with numerical solution in three dimensions, and Ikbal et al. [7] have worked on unsteady response of non-Newtonian blood flow in magnetic field without considering periodic body acceleration. Khler et al. [8] studied the wall shear stress with the help of magnetic resonance imaging (MRI) measurements of the velocity field and compared them with simulation outputs. Stroud et al. [9] have studied a 2D plaque model using modeling and simulation while Fischer et al. [10] worked on numerical method for the computational study of arterial blood flow with turbulence. The asymmetric flows in a symmetric sudden expansion channel have been studied using experimental and numerical techniques by Fearn et al. [11] and Durst et al. [12]. Mahapatra et al. [13] investigated unsteady laminar separated flow through constricted channel using finite difference technique in staggered grid distribution and suggested that the critical value of Reynolds number depends on the area reduction and the length of the constriction. Chakravarty and Sannigrahi [14] solved blood flow model with body acceleration but they do not consider the nonlinear terms in the model. Blood shows a non-Newtonian behaviour at low shear rates in tubes of smaller diameters, and Taylor [15] suggested that at high shear rates commonly found in larger arteries blood behaves like a Newtonian fluid.

With the above motivation in our mind we have worked on numerical simulations of nonlinear pulsatile unsteady Newtonian blood flow in a rigid cylindrical tube through cosine-shape stenosis under the influences of periodic body acceleration. It appears that a few studies address the issue of that nonlinear terms present in Navier-Stokes equation govern blood flow with periodic body acceleration associated with an atherosclerotic plaque. The numerical solutions are obtained of the nonlinear model using appropriate finite difference method. A comparison of the axial and radial velocities, wall shear stresses, flux rates, and the streamlines with other existing model [7] has been studied also.

## 2. Physical Assumptions and Mathematical Model

The segment of the artery is modeled as an axisymmetric cylindrical tube with radius *r*_0_. The blood is modeled as a homogeneous incompressible viscous unsteady Newtonian fluid of density *ρ* and kinematic viscosity *ν*. Therefore, the blood flow is governed by the incompressible Navier-Stokes equation.

**Figure 1:**
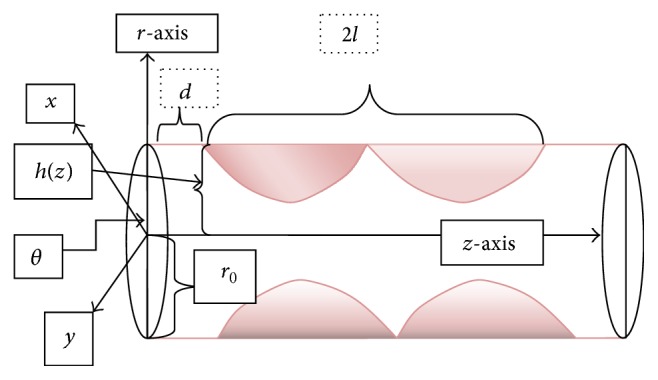
Cylindrical coordinate system with multiple stenosis along the axial direction, where 2*l* is the length of the stenosis and *d* is distance of stenosis from the radial axis.

A cylindrical coordinate system (*r*, *θ*, *z*) is chosen, where (*r*, *θ*) is the coordinate in the radial and circumferential direction, while *z* is taken along the axis of artery as shown in Figure 1. The velocity components in the axial and radial directions are *u* and *v*, respectively. The flow is driven by a prescribed dimensional oscillatory axial pressure gradient given by [16]

(1) $$\begin{array}{c} -\frac{\partial p}{\partial z}=p_{0}+p_{1}\cos\left(\;\omega t\;\right),\;t>0, \end{array}$$

where *p*_0_ and *p*_1_ are the steady component of the pressure gradient and pulsatile component, respectively. The frequency *ω* = 2*πf*, and   *f* is the heart pulse frequency. The pressure gradient in the radial direction is negligibly small as the lumen radius of artery is small compared to pressure wave length so that ∂*p*/∂*r*≃0. Also because of the human body acceleration, the axial flow is subject to an external force *F*_ext_. For the present model, we consider the periodic acceleration force given by

(2) $$\begin{array}{c} F_{ext}=a_{0}\cos\left(\;\omega t+\phi \;\right), \end{array}$$

where *a*_0_ is the amplitude of the pulse.

According to the above assumptions, the blood flow dynamics is governed by the equation of continuity

(3) $$\begin{array}{c} \frac{\partial u}{\partial z}+\frac{v}{r}+\frac{\partial v}{\partial r}=0, \end{array}$$

the momentum equation in the radial direction (flow velocity *v*)

(4) ∂v∂t=−u∂v∂z+v∂v∂r−∂p∂r+1Re∂2v∂r2+1r∂v∂r+∂2v∂z2−vr2,

and the axial direction (flow velocity *u*)

(5) ∂u∂t=−v∂u∂r+u∂u∂z−∂p∂z+1Re∂2u∂r2+1r∂u∂r+∂2u∂z2+Fext.

In (4) and (5), Re = *r*_0_ *u*_*∞*_ *ρ*/*ν* is the Reynolds number and *u*_*∞*_ is the average velocity of the blood.

**Figure 2:**
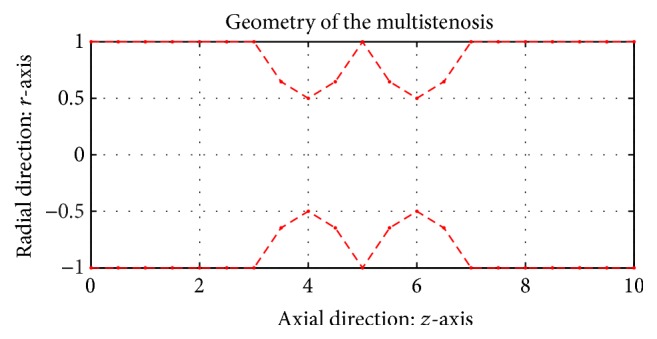
The schematic representation of the stenosis along the axial direction as given by *h*(*z*). The geometrical parameters are as follows: *l* = 2.00, *d* = 3.00, and *λ* = 0.500.

Finally, to model multiple axisymmetric stenosis, we define the following mathematical function *h*(*z*):

(6) $$\begin{array}{c} h\left(\;z\;\right)=\left\{\begin{array}{cc} 1-\lambda \cos\left(\frac{\pi z}{l}\right) & d\leq z<d+l,\\ 1+\lambda \cos\left(\frac{\pi z}{l}\right) & d+l\leq z\leq d+2l,\\ 1 & otherwise, \end{array}\right. \end{array}$$

where *λ* is a dimensionless constant and the geometry of this axisymmetric stenosis in the cross section of the artery is shown in Figure 2.

We numerically simulate (3)–(5) subject to the following initial condition

(7) ur,z,t=0,vr,z,t=0at  t=0

and the no-slip boundary conditions

(8) ∂ur,z,t∂r=0,vr,z,t=0at  r=0,ur,z,t=0=vr,z,tat  r=hz.

## 3. Numerical Simulation: Computational Method

We use the finite difference scheme to study the dynamics of blood flow through the cylindrical shape artery. To employ this method, first we transform our cylindrical domain into the rectangular domain by using the following radial transformation:

(9) $$\begin{array}{c} x=\frac{r}{h\left(z\right)}. \end{array}$$

Under this transformation, the equation of continuity (3) and the equations of motion in the radial direction (4) and axial direction (5), respectively, are rewritten as

(10) ∂u∂z+vxhz+1hz∂v∂x−xhz∂u∂xdhdz=0,

(11) ∂v∂t=−vhz∂v∂x+u∂v∂z−xuhz∂v∂xdhdz+1Re1h2z∂2v∂x2+1x∂v∂x−vx2+∂2v∂z2−1Re2xhzdhzdz∂2v∂x∂z+xhz∂v∂xd2hdz2−dh/dzhz22x∂v∂x+x2∂2v∂x2,

(12) ∂u∂t=−vhz∂u∂x+u∂u∂z−xuhz∂u∂xdhdz−∂p∂z+1Re1h2z∂2u∂x2+1x∂u∂x+∂2u∂z2−1Re2xhzdhzdz∂2u∂x∂z+xhz∂u∂xd2hdz2−dh/dzhz22x∂u∂x+x2∂2u∂x2+Fext.

Initial condition (7) and no-slip boundary condition (8) due to radial transformation (9) then become

(13) ux,z,t=0,vx,z,t=0,at  t=0,∂ux,z,t∂x=0,vx,z,t=0at  x=0,ux,z,t=0=vx,z,tat  x=1.

Let us first apply the finite difference discretization scheme to solve nonlinear model equations (10)–(12). We use the central difference approximation to discretize the spatial derivatives and the explicit forward finite difference approximation to discretize the time derivative in the following manner:

(14) ∂u∂z=ui+1,jn−ui−1,jn2Δz,∂2u∂z2=ui+1,jn−2ui,jn+ui−1,jnΔz2,∂u∂x=ui+1,jn−ui−1,jn2Δx,∂u∂t=ui,jn+1−ui,jnΔt,

where

(15) ui,jn=uxj,zi,tn,zi=i−1Δz,i=1,2,…,M+1,xj=j−1Δx,j=1,2,…,N+1,tn=n−1Δt,n=1,2,….

Similarly we approximate all the partial derivatives of *v*.

The axial velocity (*u*)_*i*,*j*_^*n*^ is obtained from (10) and (12) by applying the above finite difference scheme at any point (*z*_*i*_, *x*_*j*_) in the domain of interest at any time *t*_*n*_ with the help of the following discretize initial and boundary conditions (discretization of (13)):

(16) ui,j1=0,vi,j1=0,ui,1n=ui,2n,vi,1n=0,ui,N+1n=vi,N+1n=0,

subject to the input pressure gradient and external force *F*_ext_ from relations (1) and (2). The radial velocity (*v*)_*i*,*j*_^*n*^ is obtained from (10) and (11).

Finally, we determine the volumetric flow rate

(17) $$\begin{array}{c} Q=2\pi \overset{h}{\underset{0}{\int }}ru\;dr=2\pi h{\left(\;z\;\right)}^{2}\overset{1}{\underset{0}{\int }}xu\;dx \end{array}$$

and the wall shearing stress

(18) $$\begin{array}{c} \tau ={\left.\;-\mu \frac{du}{dr}\;\right|}_{r=h\left(z\right)}=-\frac{\mu }{h\left(z\right)}\frac{du}{dx} \end{array}$$

in the rectangular domain with the help of transformation (9), where *μ* is the viscosity. The discretize version of *Q* and *τ* is given by the following equations:

(19) Qin=2πhin2∫01xjui,jndxj,τin=−μhinui,N+1−ui,NΔx.

## 4. Simulation Results and Discussions

In this section, we shall discuss the numerical simulation of the nonlinear equations to study the influence of stenoses and body acceleration on the blood flow for different values of the physical parameters. The simulation parameters are as follows [7, 14]: *l* = 2.00, *d* = 3.00, *λ* = 0.5, Re = 400, 600, and 800, *p*_0_ = 0.1, and *p*_1_ = 0.2 × *p*_0_. The results obtained for axial velocity by solving explicit finite difference scheme with various grid sizes are taken in order to achieve the convergence and stability. We perform the experiments for grid size 60 × 60 and 100 × 100 with *dt* = 0.01 and 0.001. The results are found to be very similar in both cases.

**Figure 3:**
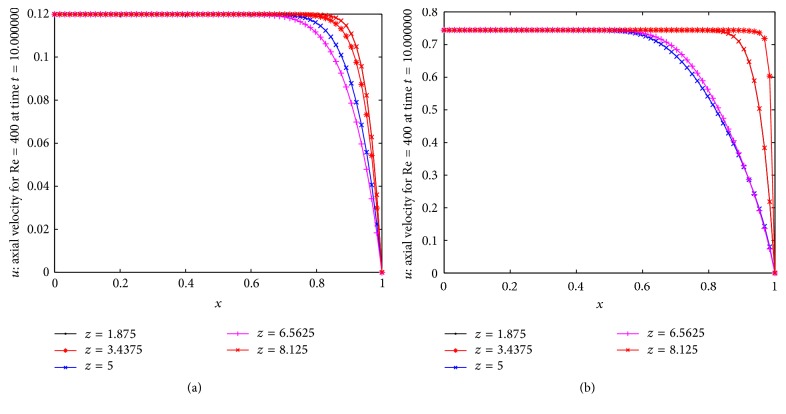
Distribution of axial velocities at different *z* for Re = 400. Axial velocity (a) without body acceleration and (b) with body acceleration.

**Figure 4:**
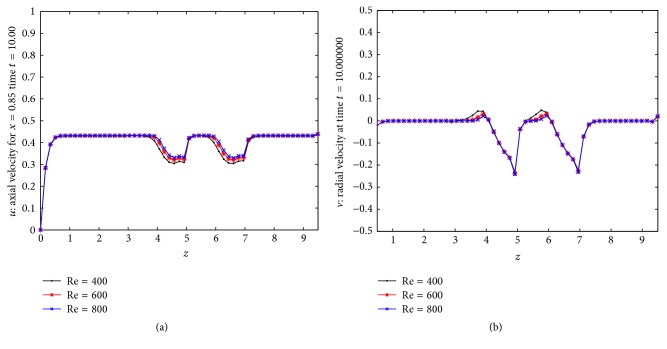
Distribution of (a) axial velocities for various Reynolds numbers and (b) radial velocities for different Reynolds number.

Figures 3(a) and 3(b) represent the behavior of the axial velocity profile of the blood at time *t* = 10 without and with body acceleration, respectively. Both figures are drawn for Re = 400 at different *z*. The comparative study between the figures (Figures 3(a) and 3(b)) reveals that the body acceleration enhances the axial velocity. The curves in these two figures reveal that the velocity profile is constant for 0 ≤ *x* ≤ *x*_*m*_ and then velocity decreases and finally goes to zero on the constricted arterial wall. The values of *x*_*m*_ depend on both the body acceleration and the stenosed zone. Also in the stenosed zone of the artery (3 ≤ *z* ≤ 7), the velocity is low and in presence of body acceleration, the velocity sharply decreases in this stenosed zone.  Figure 4(a) shows the results for the distribution of axial velocity over the stenosed artery for three different Reynolds numbers. We can say from this figure that as *z* enters into the stenosed zone the axial velocity starts decreasing from its maximum value in nonstenosed zone until the first constriction attaint its maximum value near *z* = 4 and then it gradually increases up to *z* = 5.2 and again it starts decreasing till the maximum height of the second stenosis and gradually starts increasing thereafter and finally again flows with maximum velocity in nonstenosis region. The three curves here indicate that the axial velocity increases in the constricted part of the artery as Reynolds number increases under the influence of body acceleration.  Figure 4(b) represents the results for the distribution of radial velocity in the multiple stenosed artery for three different Reynolds numbers. From the figure we can say that the direction of radial velocity is negative in the stenosis zone due to presence of multiple stenosis. Thus the multiple stenosis and the Newtonian characteristics of the flowing blood affect the axial velocity profile which can be estimated by the relevant curves of the present figure.

**Figure 5:**
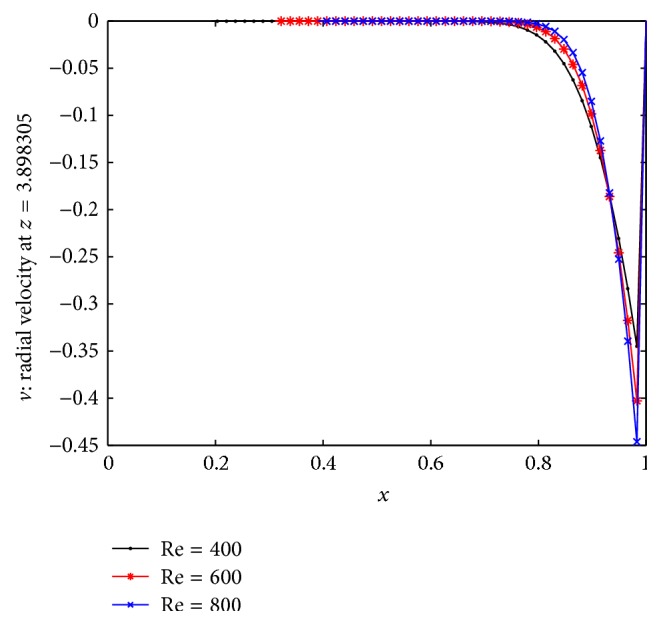
The distribution of radial velocity at *z* = 3.9.

The curves in Figure 5 describe the nature of radial velocity for three different Reynolds numbers. The velocity initially starts with zero and continues till *x* = 0.8 and then it decreases gradually in negative direction.

**Figure 6:**
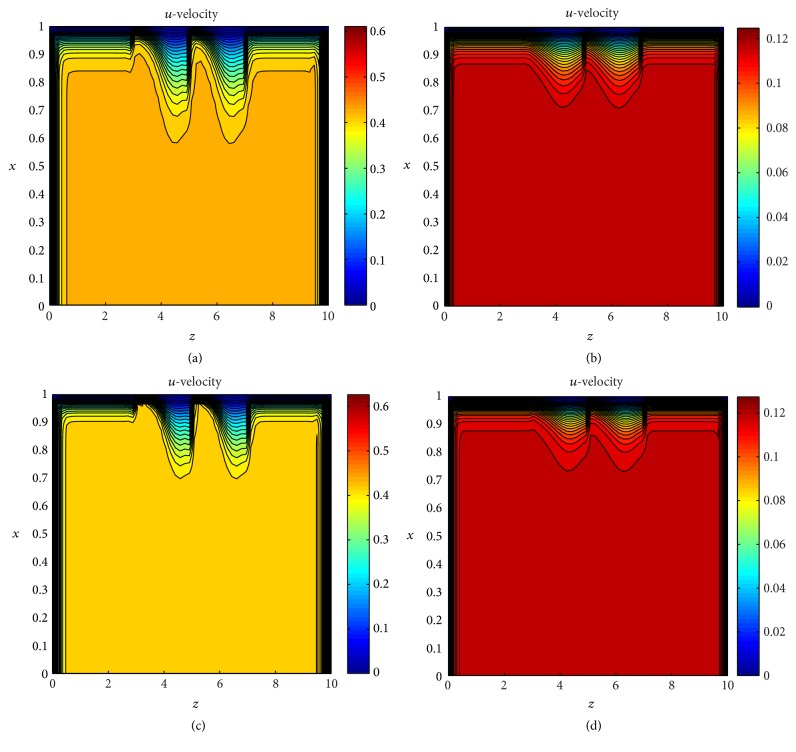
Contour distribution of (a) axial velocities for Reynolds number Re = 400 with body acceleration, (b) axial velocities for Re = 400 without body acceleration, (c) axial velocities for Reynolds number Re = 800 with body acceleration and (d) axial velocities for Re = 800 without body acceleration.

**Figure 7:**
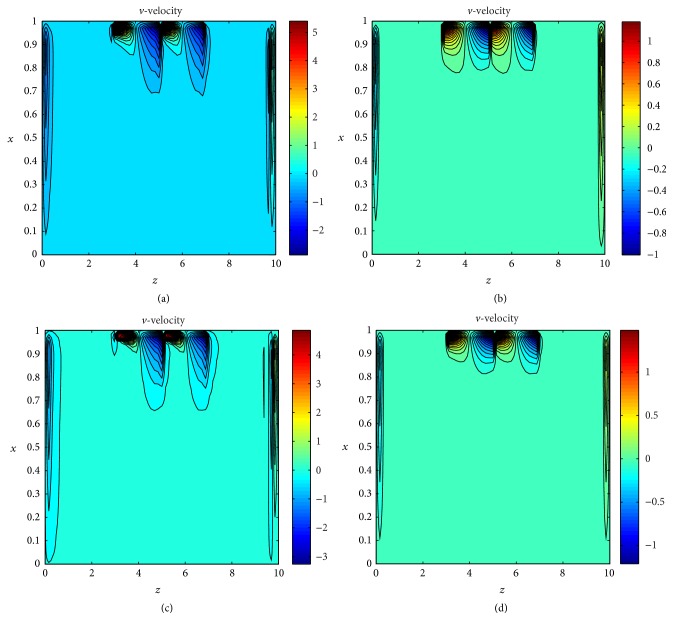
Contour distribution of (a) radial velocities for Reynolds number Re = 400 with body acceleration, (b) radial velocities for Re = 400 without body acceleration, (c) radial velocities for Reynolds number Re = 800 with body acceleration, and (d) radial velocities for Re = 800 without body acceleration.

**Figure 8:**
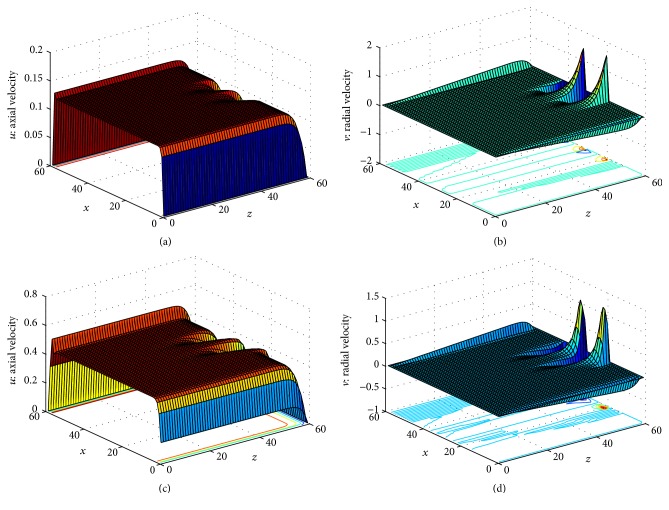
3D plot of (a) axial velocities without body acceleration at Re = 400, (b) radial velocities without body acceleration at Re = 400, (c) axial velocities with body acceleration at Re = 400, and (d) radial velocities with body acceleration at Re = 400.

To test the effects of body acceleration on axial and radial velocities profile several simulations have been carried out using the contour plot as shown in Figures 6(a)–6(d) and Figures 7(a)–7(d) respectively. The velocity profiles *u* and *v* are shown in different region with different colors representing the value of velocity with the help of color bar. One can see from these plot that velocity profile is divided into different layers due to the constriction of the artery and changes in the plot also can be observed in case of no body acceleration. We have shown the distribution of axial and radial velocities in Figures 8(a) and 8(d) in entire upper half segment of the artery using 3D plot.  Figure 8(a) shows the axial velocity profile of the flow without body acceleration at time *t* = 10 for Re = 400. One can see that the velocity profile is constant in the nonstenosed zone and varies over the constricted area. In case of body acceleration the profile is shown in Figure 8(c).

**Figure 9:**
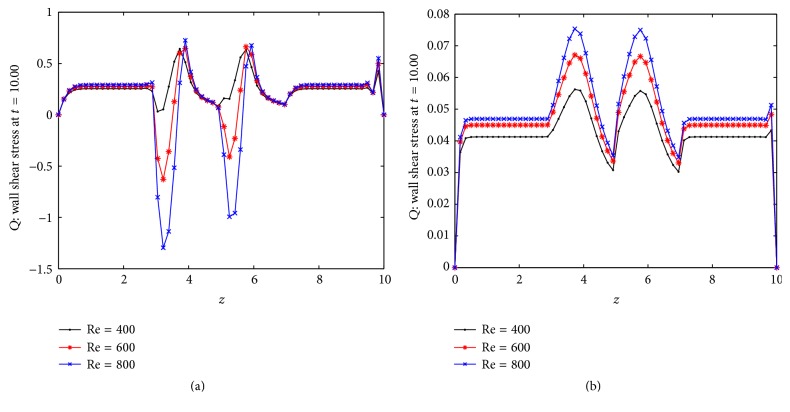
Distribution of (a) wall shear stress with body acceleration and (b) wall shear stress without body acceleration.

**Figure 10:**
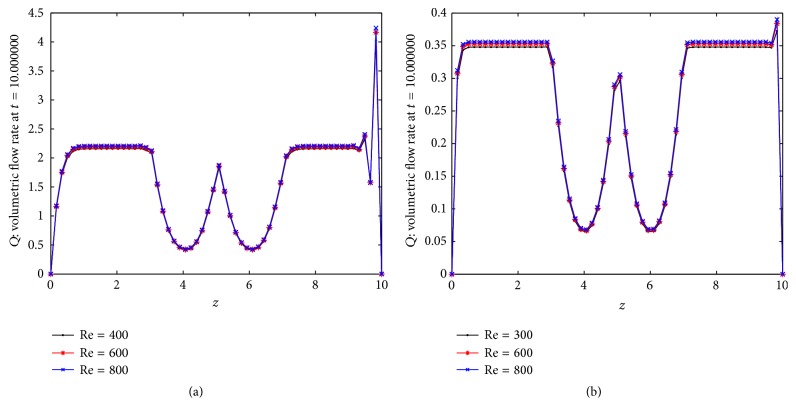
Distribution of (a) flux with body acceleration and (b) flux without body acceleration.

Wall shear stress plays an important role in the creation and propagation of arteriosclerosis. If the wall shear stress is high then it may damage the arterial wall and is the main cause of the intimal thickening. On the other hand, the plaque formation in an artery is created in the regions of low arterial wall shear stress. Atherosclerotic lesions are associated with low and high wall shear stress. So it is important to study the wall shear stress distribution in the multistenosed artery. Figures 9(a) and 9(b) show the distribution of wall shear stress on the arterial segment for three different Reynolds numbers. The wall shear stress increases rapidly near to the peak of the constriction. Here the effects of Reynolds number can be observed from the figure. The wall shear stress increases as Reynolds number increases. Figures 10(a) and 10(b) show the distribution of flux over the stenosed artery for different Reynolds number. One can conclude that flux decreases near the picks of the stenosis.

**Figure 11:**
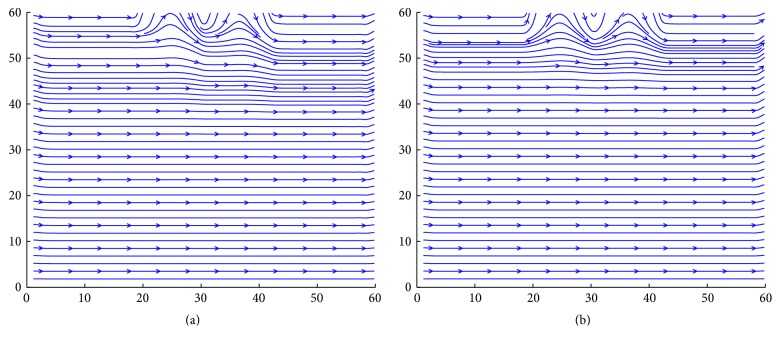
Distribution of (a) streamlines with body acceleration and (b) streamlines without body acceleration at time *t* = 10 in the upper half segment of the artery.

The streamlines of the blood flow in the artery with multistenosis are found in the transformed rectangular domain with grid 60 × 60 in the upper half zone and same grid also taken for lower half portion. We have plotted the different types of streamlines in Figures 11(a) and 11(b) at Re = 400. All the streamlines follow the straight line path near the axis which gradually get perturbed more towards the wall of the stenosed artery. It is interesting to observe that several flow lines are attracted towards the stenotic wall upstream with the formation of circulation zones while others pass through the constricted region directly following the main stream.

**Figure 12:**
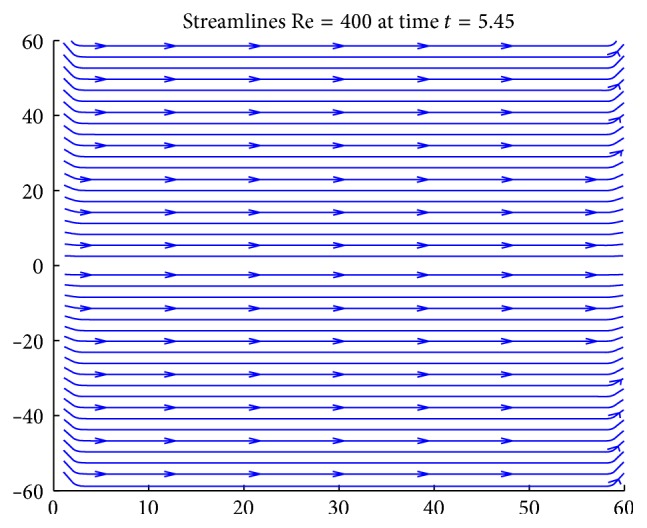
The streamline patterns of the flow at Re = 400 when there is no constriction.

Figure 12 shows the streamline patterns at Re = 400 when the artery is free from stenosis. It is observed that the lines are parallel to the axial direction.

**Figure 13:**
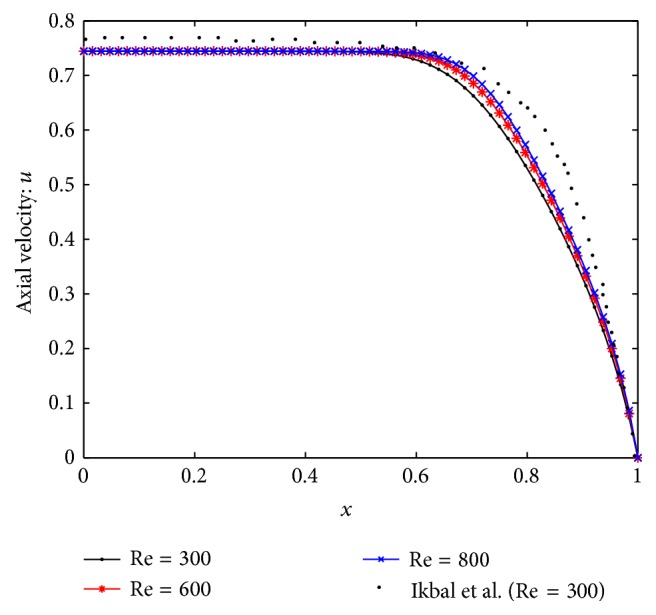
Validation of axial velocity at Re = 300.

For the purpose of model validation, the axial velocity profile is compared with [7], and as shown in Figure 13, the results are found to be in good agreement though their studies were based on the stenotic blood flow in which the streaming blood was treated as non-Newtonian fluid in magnetic field. Also the result agrees qualitatively well for Newtonian fluid with Tu et al. [17].

## 5. Conclusions

A nonlinear mathematical model for blood flow in a multiple stenosed arterial segment has been developed under the influence of body acceleration. The numerical simulation of blood flow is investigated in this study. As the Reynolds number increases, the wall shear stress increases. The multiple stenosis has significant effect on the wall shear stress in such a way that it develops more at the constricted locations than all other sites of the artery. The streamline pattern shows the distinct boundary layer characteristics in the arterial segment. This is validated by the flow visualisation observed by the simulation studies of [7]. The results obtained here would help researchers greatly in gaining better insight into blood flow models through the multistenosis artery under the influence of body acceleration.
